# Case Report: Surgery for proximal gastric cancer with splenic artery aneurysm: approach and complication mangement

**DOI:** 10.3389/fonc.2025.1614556

**Published:** 2025-07-18

**Authors:** Xuerui Shu, Fu Xiang, Xuedong Xu, Lin Jing, Riyu Tian, Yiao Xia

**Affiliations:** The First Affiliated Hospital of Dalian Medical University, Department of General Surgery, Dalian, Liaoning, China

**Keywords:** laparoscopic proximal gastrectomy, spleen preservation, short gastric vessels, gastric tube, proximal gastric cancer, Roux-en-Y gastrojejunostomy

## Abstract

**Introduction:**

Gastric cancer surgery is gradually shifting from extensive, standardized operations to individual, precise procedures. The two surgical options for proximal gastric cancer are total gastrectomy and proximal gastrectomy. In recent years, proximal gastrectomy has gained wider acceptance in clinical practice, with increasingly relaxed indications. The present report describes an extremely rare clinical case of proximal gastric cancer.

**Case presentation:**

A 64-year-old man with proximal gastric cancer after splenic artery aneurysm embolization underwent proximal gastrectomy with preservation of the short gastric vessels to retain the spleen. Despite the difficult treatment process, the patient achieved a favorable outcome at the 10-month follow-up.

**Conclusion:**

This report provides a valuable reference for the individualized treatment of proximal gastric cancer, highlighting the importance of considering potential complications such as external pressure from anatomical anomalies in the postoperative management of gastric cancer.

## 1 Introduction

Globally, gastric cancer is the fifth most common cancer and fourth leading cause of cancer-related deaths, with a high incidence rate (1). Since total gastrectomy was first proposed in the 1970s, studies have shown that total gastrectomy (TG) and proximal gastrectomy (PG) have similar survival rates (2). In recent years, owing to the continuously increasing prevalence of proximal gastric cancer (PGC) (3), a deeper understanding of the patterns of lymph node metastasis, and the emergence of anti-reflux surgical techniques, PG has gradually been recognized as a function-preserving surgery in clinical practice. This report presents an extremely rare case of proximal gastric cancer with preservation of the short gastric arteries.

## 2 Case presentation

**Figure 1:**
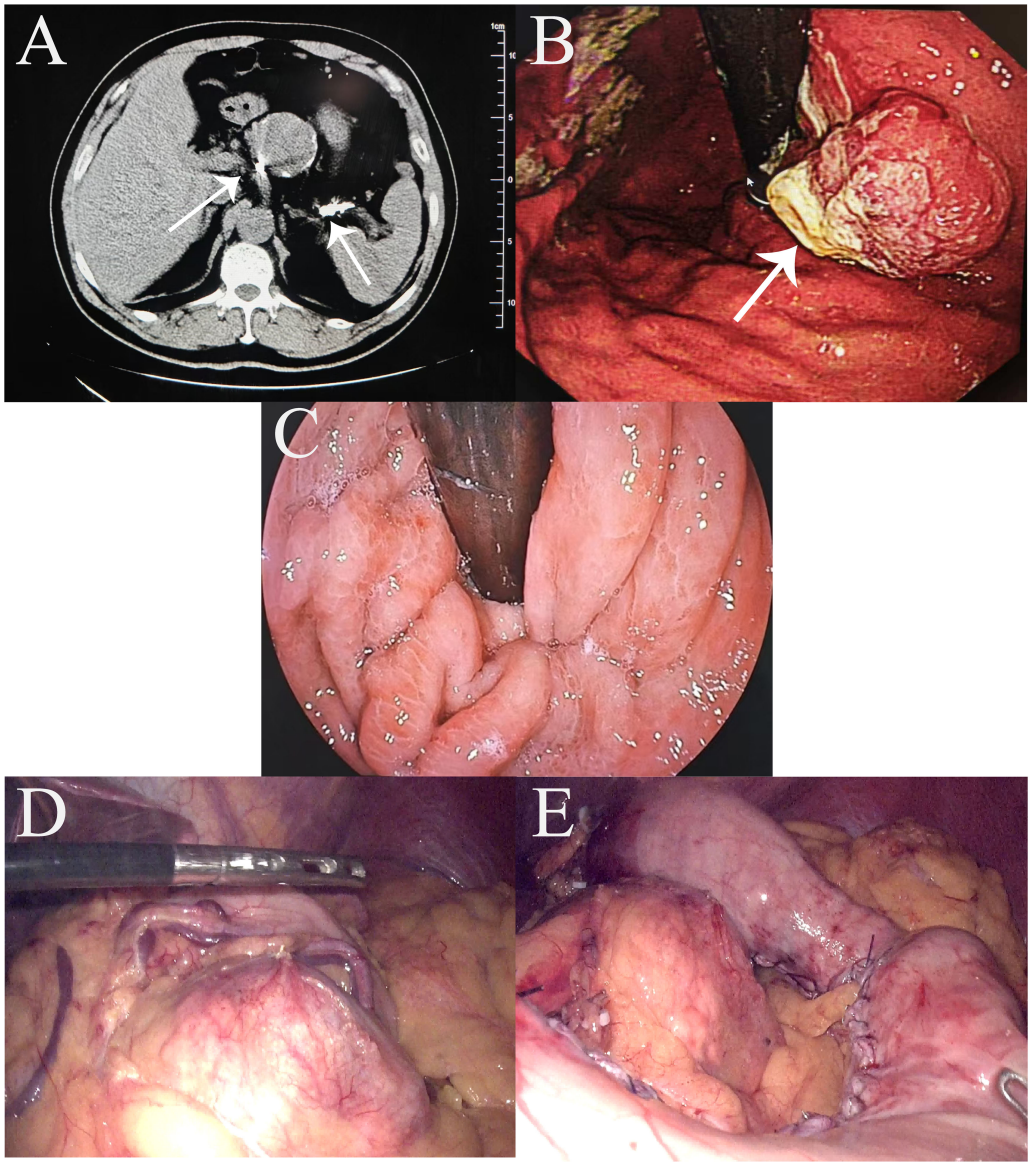
Preoperative findings. **(A)** Abdominal enhanced computed tomography scan showing embolic material (arrows) at the proximal and distal ends of the splenic artery; **(B)** Preoperative gastroscopy. **(C)** Intraoperative gastroscopy successfully passed through the gastric tube. **(D)** Right gastroepiploic vessels with compensatory dilatation; splenic artery aneurysm visualized. **(E)** Images after first proximal gastrectomy.

**Figure 2:**
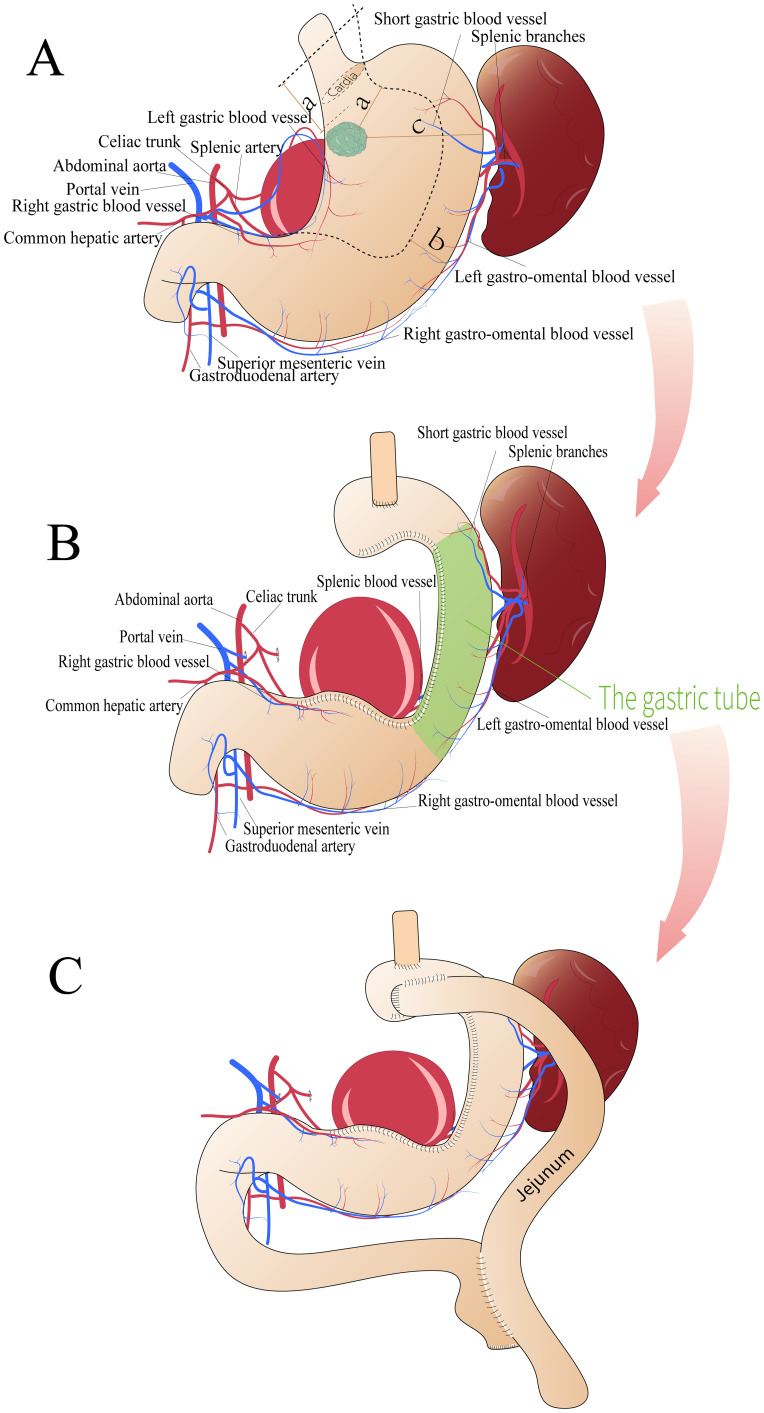
Surgical schematic diagram. **(A, B)** First surgical plan showing (a) the shortest distance between the tumor edge and the end of the incision (>5 cm), (b) the reserved stomach wall width (>3 cm), and (c) the shortest distance between the tumor edge and the greater curvature side (10 cm). **(C)** Second surgical plan.

A 64-year-old man presented with upper abdominal pain for 6 months, which had worsened in the month prior to presentation, and was admitted to our center. His relevant medical history included splenic artery aneurysm embolization. An abdominal computed tomography (CT) scan revealed the following: (1) post-surgical changes from splenic artery aneurysm clipping (**Figure 1A**) and (2) a slightly thickened gastric wall. Gastroscopy revealed a protruding approximately 3 cm lesion, located 2 cm distal to the cardia on the posterior wall of the lesser curvature (**Figure 1B**). The preoperative diagnosis was poorly differentiated adenocarcinoma of the upper stomach (cT2N0M0). Intraoperative laparoscopic exploration revealed no tumor invasion through the anterior wall of the upper gastric body. Greater curvature/short gastric vessels dilated. 7-cm splenic artery aneurysm (1 cm from proximal splenic artery). Firm embolic materials at splenic artery root/distal end (**Figure 1D**). Standard D2 gastrectomy requiring ligation of perigastric vessels would compromise splenic perfusion, risking infarction/abscess. Given this and the prohibitive difficulty of laparoscopic splenectomy due to the giant aneurysm, we preserved the spleen with its greater curvature gastric segment and intact vascular arcade. Therefore, a 3-dimensional (3D) laparoscopic PG with esophagogastrostomy and perigastric lymph node dissection was performed (**Figure 1E**) (**Figures 2A, B**). The procedure went smoothly, and the endoscope was passed through the gastric tube without difficulty, demonstrating excellent patency (**Figure 1C**). Intraoperative and postoperative contrast-enhanced CT showed good splenic blood supply.

**Figure 3:**
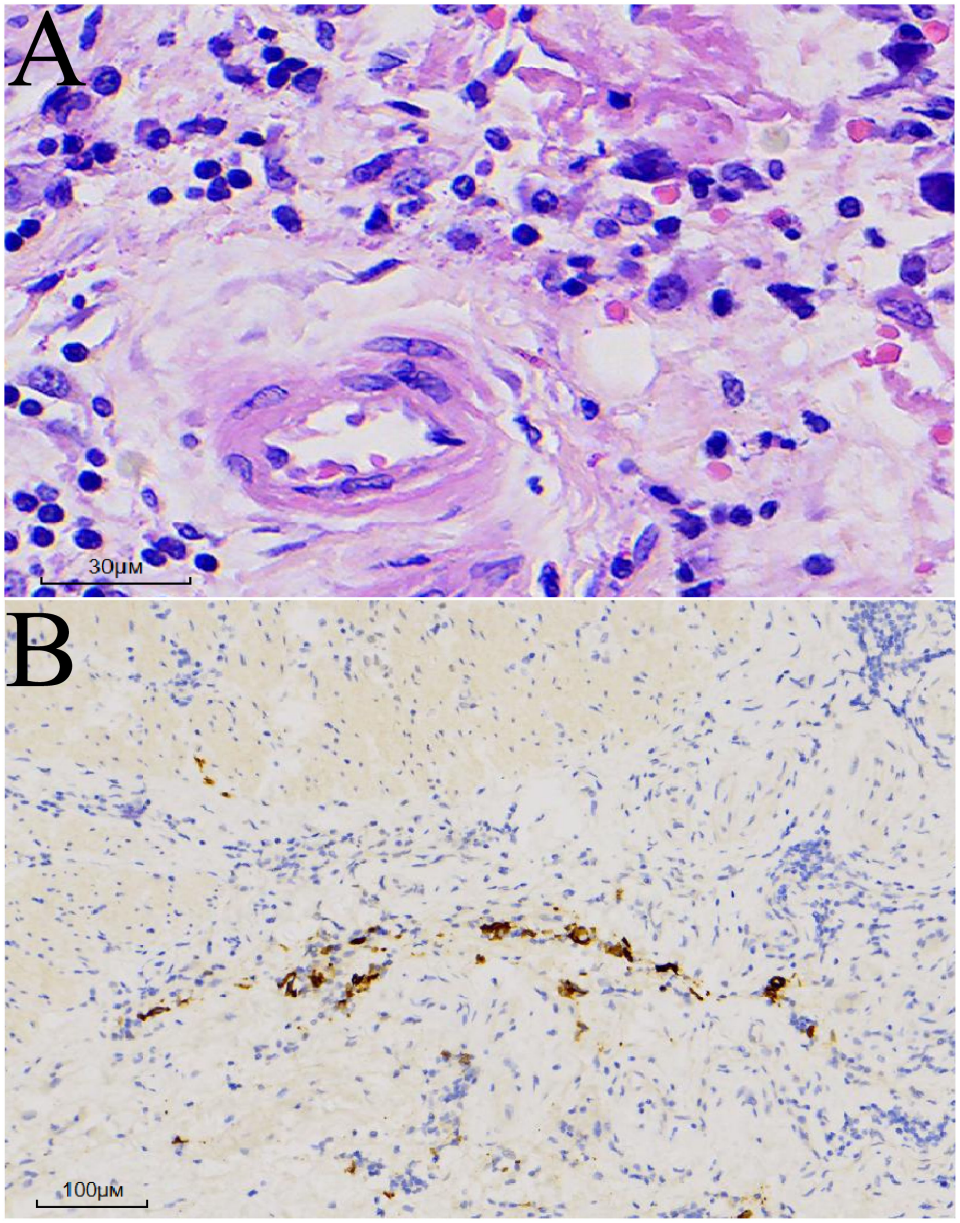
Postoperative pathology. **(A)** Hematoxylin and eosin (HE) staining: Atypical cells exhibiting diffuse infiltrative growth. **(B)** Cytokeratin (CK) immunostaining: Positive.

The postoperative pathological report revealed a poorly differentiated adenocarcinoma measuring 3.3 × 3 × 0.4 cm. Immunohistochemical results indicated focal neuroendocrine differentiation, focal invasion into the muscularis propria, and no neurovascular invasion (**Figure 3**). No tumor involvement was observed at the proximal and distal margins. Examination of 17 lymph nodes (stations 1, 2, 3, 7, and 8a) revealed no metastasis (0/7, 0/2, 0/5, 0/2, 0/1 respectively). Further immunohistochemical findings were as follows: CK(+) in specimens 01-04, ARGINASE-1(-), BRG1(+), BRM(+), CD10(-), CD56(-), CDX-2(-), CgA(-), CK20(-), CK5/6(-), CK7(focal+), CK8/18(+), EBER(-), GPC-3(-), HepPar-1(-), INI1(+), INSM1(focal weak+), p40(-), SALL4(-), Syn(focal+), GST-P(+), Her-2(0), Ki-67(80%+), MLH1(loss), MSH2(retained), MSH6(retained), p53(wild-type), PMS2(loss).

**Figure 4:**
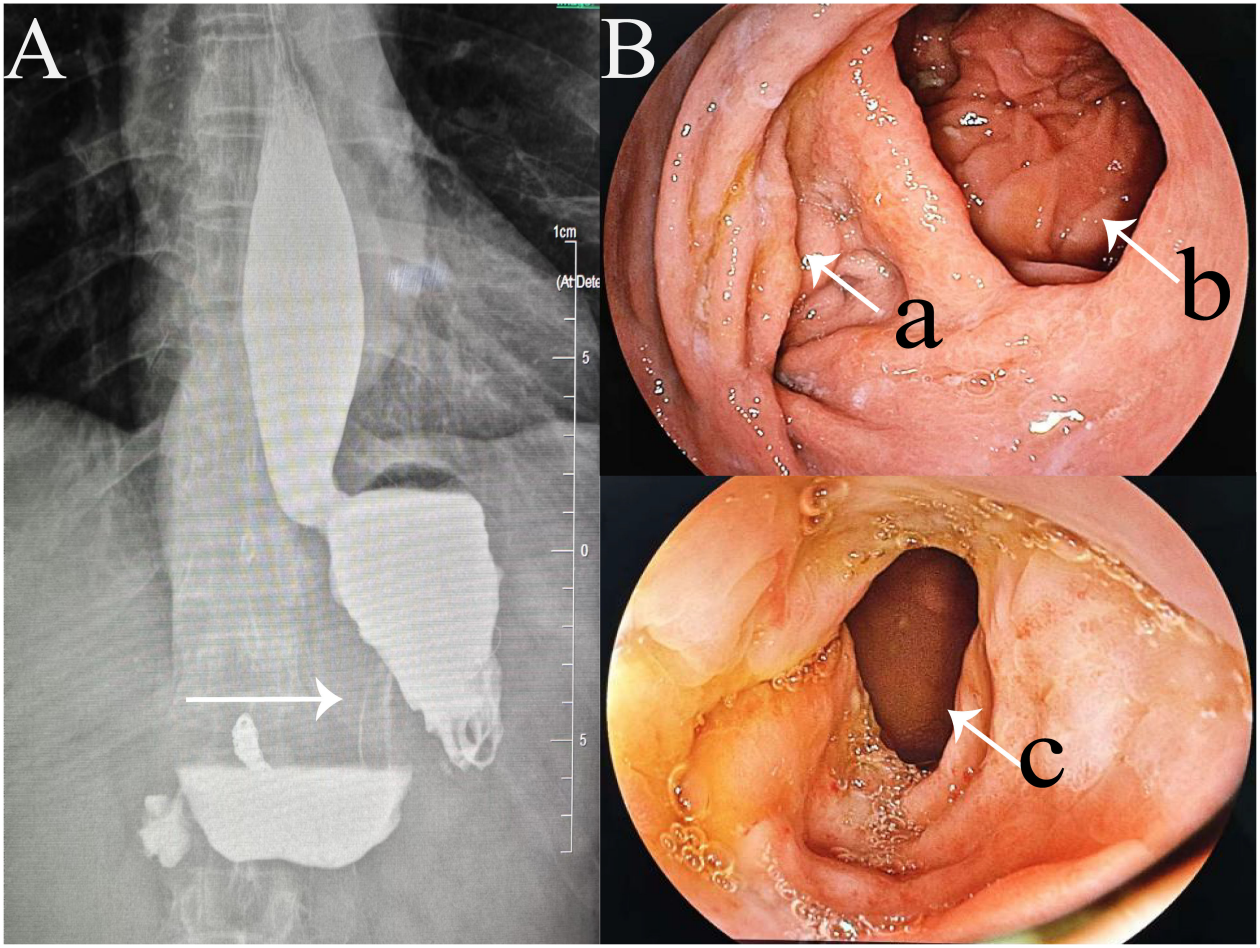
Two postoperative examinations. **(A)** Upper gastrointestinal series showing the stenotic segment of the gastric tube (arrow); **(B)** Gastroscopy images showing (a) entrance of stenotic segment of the gastric tube; (b) gastrojejunostomy anastomosis; and (c) downstream end of the stenotic segment.

On the 13^th^ postoperative day, the patient developed upper abdominal fullness and discomfort after consuming non-bloating liquids, accompanied by significant acid reflux symptoms. Follow-up abdominal CT revealed slight edema around the esophagogastric anastomosis and dilation, with fluid accumulation in the thoracic esophagus. Fasting and nutritional support failed to alleviate symptoms. An upper gastrointestinal series revealed partial obstruction of the residual stomach (**Figure 4A**), located at the angular incision of the gastric tube. However, the contrast agent was able to pass through the gastric tube, albeit slowly. Gastroscopy revealed edema of the gastric tube and gastric lumen stenosis with good peristalsis, effectively ruling out the possibility of gastric paraplegia syndrome. Significant external pressure was noted on the endoscope while passing through the gastric lumen near the splenic artery aneurysm, resulting in a slight increase in the pressure required to advance the endoscope. Therefore, postoperative gastric obstruction due to external pressure from the splenic artery aneurysm was considered. One month postoperatively, a 3D laparoscopic Roex-en-Y anastomosis of the jejunum to the gastrojejunostomy, with a jejunojejunostomy was performed, converting to a double-pathway approach to alleviate gastric lumen stenosis (**Figure 2C**). On the 13^th^ day after the second surgery, the patient was discharged smoothly.

One year postoperatively, the patient was assessed with enhanced CT of the upper abdomen and gastroscopy (**Figure 4B**), showing good patency of both the gastric tube and gastrojejunal anastomosis.

## 3 Discussion

Two surgical options exist for proximal gastric cancer: TG and PG. According to the 6^th^ edition of the Japanese Gastric Cancer Treatment Guidelines, TG is recommended for T2 or higher proximal gastric cancer (4). However, some researchers argue that PG is indicated for T2 tumors smaller than 4.0 cm (5). In the present case, the widest tumor diameter was 3 cm, located on the posterior wall of the upper part of the gastric body, 2 cm away from the cardia, and 10 cm from the greater curvature, making either TG or PG feasible. However, as the patient had a splenic artery aneurysm and embolization of the main trunk of the splenic artery, performing a standard PG or TG procedure would sever the blood supply to the spleen, requiring concurrent splenectomy. However, as the patient had a splenic artery aneurysm and embolization of the main trunk of the splenic artery, performing a standard PG or TG procedure would sever the blood supply to the spleen, requiring concurrent splenectomy. Sano et al. evaluated the role of splenectomy in PG for proximal gastric cancer and concluded that splenectomy should be avoided in cases where cancer does not invade the greater curvature (6). Since then, some studies have statistically analyzed the clinical effects of prophylactic splenectomy on advanced proximal gastric cancer, indicating that splenectomy should be avoided unless the tumor directly invades the spleen (7). In addition, a Japanese multicenter retrospective analysis demonstrated that when the tumor was distal to the cardia by 3 cm or less, the lymph node metastasis rate in the greater curvature side and the pyloric region was low (<2.2%) (8). Meanwhile, considering the patient’s specific conditions, the splenectomy poses certain challenges: First, due to the history of splenic artery embolization, preoperative CT reveals a splenic artery aneurysm adjacent to the aortic root, with embolization coils visible at both proximal and distal ends. Transection at the proximal end would entail high intraoperative bleeding risk and difficult suturing; transection at the distal end requires dissection near the splenic hilum, which is technically challenging due to poor exposure. Additionally, long-term stent placement has increased tissue friability, posing a risk of detachment and tearing. The splenic artery aneurysm further obscures the surgical field, raising the likelihood of conversion to laparotomy. Second, if total gastrectomy combined with splenectomy is chosen, the subsequent anastomosis between the small intestine and esophagus may face excessive tension due to the location and size of the splenic artery aneurysm, heightening the risk of postoperative anastomotic leakage. In the present report, PG was successfully performed while preserving the vascular arch on the side of the greater curvature. This approach not only achieved the goal of lymph node dissection (0/17) but also avoided the risks associated with splenectomy. Splenectomy patients have higher adjusted short- and long-term mortality risks than the general population (9). Perform splenectomy only after carefully weighing risks against potential benefits.For this patient, as splenic hilar lymph nodes showed no metastasis and the family requested spleen preservation, the surgeon decided to spare the spleen.

In the present case, during the initial surgery, the patency of the gastric tube was confirmed using a gastroscope. However, postoperative gastric lumen obstruction developed owing to a combination of tissue edema and external compression from the splenic artery aneurysm. Notably, the angular incision is the most common site of obstruction in gastric tubes (10). Management of stenosis includes observation, endoscopic intervention, stenoplasty, and conversion surgery. In our patient, neither observation nor attempts to reduce edema were effective, and the splenic artery aneurysm external pressure was believed to be the cause of the obstruction. Given the high probability of recurrence after endoscopic dilation, the only approach to ensure healing was to proceed with gastric bypass surgery using a dual-access surgical method (11). Moreover, the tumor size was 3 cm, and to ensure an adequate surgical margin, the width of the tubular stomach was sacrificed. Research suggests that gastric tube reconstruction with a width of 3 to 6 cm is feasible (12) and in this case, the gastric tube width was 3 cm, which was the primary factor considered for the secondary surgery. Therefore, this surgical approach is the first attempt in such cases, the optimal ratio of marginal distance and residual stomach width is unknown, and further studies are needed. To minimize the need for a second surgery, more stringent recommendations should be established based on the primary lesion size and staging. While the width of the conduit is discussed as a factor, increasing its width would result in a shorter tumor resection margin. Depending on the tumor’s location, depth and pattern of spread, creating a gastric conduit can be technically challenging. Therefore, this may be a procedure with limited indications.

At the last follow-up, the patient was able to consume regular food without experiencing symptoms of acid reflux. Moreover, the previously narrowed gastric tube was confirmed to be fully patent using endoscopy. However, owing to the short follow-up period, long-term monitoring is essential to determine the overall prognosis. This report provides a valuable reference for similar cases.

## 4 Conclusion

This case report provides a valuable reference for the individualized treatment of proximal gastric cancer requiring radical resection to preserve the gastric short vessels after splenic artery embolization.

## Data Availability

The original contributions presented in the study are included in the article/**Supplementary Material**. Further inquiries can be directed to the corresponding author.
