# Supplementary figures and images for: Case Report: Surgery for proximal gastric cancer with splenic artery aneurysm: approach and complication mangement
Source: Front Oncol. 2025 Jul 18;15:1614556. doi: 10.3389/fonc.2025.1614556 (PMC12313472; doi:10.3389/fonc.2025.1614556)

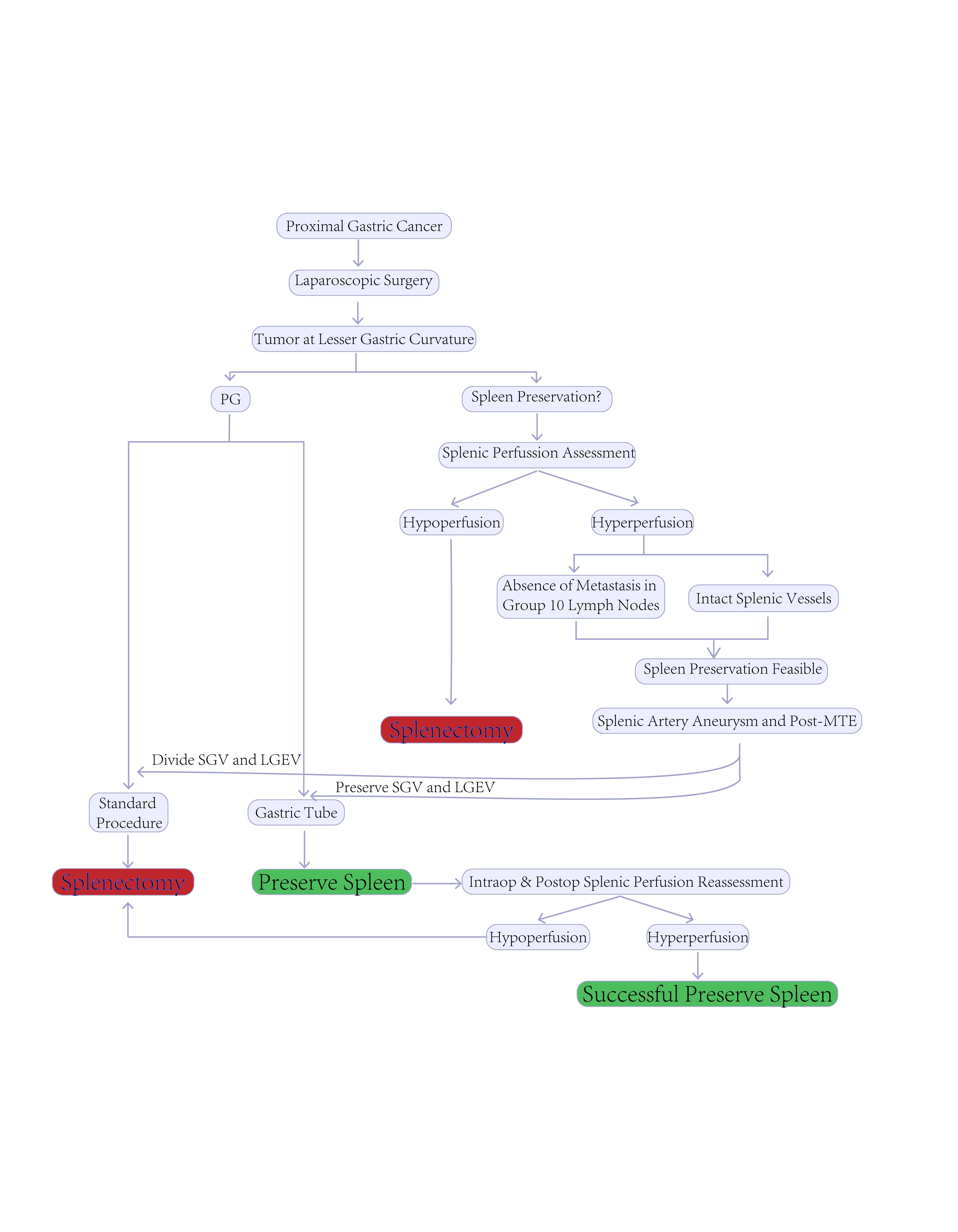

Supplement: Supplementary file 1 [file Image1.tif]
